# Re‐defining the clinicopathological spectrum of neuronal intranuclear inclusion disease

**DOI:** 10.1002/acn3.51189

**Published:** 2020-09-15

**Authors:** Hao Chen, Likui Lu, Bin Wang, Guiyun Cui, Xingqi Wang, Yujing Wang, Hafiz Khuram Raza, Yan Min, Keke Li, Yingying Cui, Zhigang Miao, Bo Wan, Miao Sun, Xingshun Xu

**Affiliations:** ^1^ Department of Neurology the Second Affiliated Hospital of Soochow University Suzhou City 215004 China; ^2^ Institute for Fetology the First Affiliated Hospital of Soochow University Suzhou City 215006 China; ^3^ Department of Neurology the Affiliated Hospital of Xuzhou Medical University Xuzhou City 221600 China; ^4^ Department of Neurology the First Affiliated Hospital of Soochow University Suzhou City 215006 China; ^5^ Institute of Neuroscience Soochow University Suzhou City 215123 China; ^6^ School of Life Science Jiangsu Normal University Xuzhou City 221600 China; ^7^ Department of Pathology Tongshan County Hospital of Traditional Chinese Medicine Xuzhou City 221600 China; ^8^ Department of Imaging the Affiliated Hospital of Xuzhou Medical University Xuzhou City 221600 China; ^9^ Department of Pathology the Affiliated Hospital of Xuzhou Medical University Xuzhou City 221600 China; ^10^ Jiangsu Key Laboratory of Neuropsychiatric Diseases Soochow University Suzhou City 215123 China

## Abstract

**Background:**

The rapidly increasing case reports revealed that neuronal intranuclear inclusion disease (NIID) had concomitant other system symptoms besides nervous system symptoms. In this study, we systematically evaluated the symptoms, signs, auxiliary examination, and pathological changes in different systems in NIID patients.

**Methods:**

NIID patients were confirmed by examining GGC repeats in the *NOTCH2NLC* gene. Clinical data of NIID patients including symptoms, signs, and auxiliary examinations were collected for analysis. Ubiquitin and p62 were detected in different tissues from previous surgical samples.

**Results:**

Fifty‐one NIID patients from 17 families were included in this study. Except neurological symptoms, clinical manifestations from other systems were very notable and diverse. The proportions of different system symptoms were 88.2% in nervous system, 78.4% in respiratory system, 72.5% in circulatory system, 72.5% in locomotor system, 66.7% in urinary system, 64.7% in digestive system, 61.5% in reproductive system, and 50.0% in endocrine system. In addition, other common symptoms included sexual dysfunction (43.1%), pupil constriction (56.9%), blurred vision (51.0%), and hearing loss (23.5%). Ubiquitin and p62‐positive cells were found in different tissues and systems in 24 NIID patients with previous surgery. Initial symptoms of NIID and median onset age in different systems also revealed system heterogeneity of NIID.

**Interpretation:**

For the first time, we systematically demonstrated that NIID is a heterogeneous and systemic neurodegenerative disease by providing clinical and pathological evidence. In addition to the nervous system, the clinical symptomatic and pathological spectrum of NIID has been extended to almost all systems.

## Introduction

Neuronal intranuclear inclusion disease (NIID) was first reported in 1968 by Lindenberg *et al*. in Germany.^1^ It was considered as a rare neurodegenerative disease before eosinophilic intranuclear inclusions in skin biopsy were considered as the pathological hallmark for the diagnosis of NIID.^2^ NIID is a heterogeneous disease and is characterized with highly variable clinical manifestations including dementia, Parkinsonism, and ataxia.^3^ In addition, abnormal high‐intensity signals along the corticomedullary junction in diffusion‐weighted imaging (DWI) of magnetic resonance imaging (MRI) are regarded as a diagnostic indicator for NIID.^3^, ^4^ With the help of imaging technologies, skin biopsy and genetic advances on the *NOTCH2NLC* gene as a causative gene,^5^ NIID was no more considered as a rare disease because more and more patients were diagnosed with NIID.^5^, ^6^, ^7^, ^8^ So far, the underlying mechanisms of NIID are unclear. Post‐mortem pathological findings revealed that eosinophilic intranuclear inclusions were distributed in multiple regions in the central nervous system (CNS) including the cerebral cortex, basal ganglia, brainstem, and spinal cord.^3^ These intranuclear inclusions were ubiquitin‐positive and p62‐positive,^3^ suggesting that some non‐degraded and ubiquitinated proteins were aggregated in the nuclei. Because eosinophilic intranuclear inclusions were also found in the somatic cells, eosinophilic intranuclear inclusions in skin biopsy are considered as the pathological hallmark for the diagnosis of NIID before the genetic diagnosis was established.^5^, ^6^, ^7^

Although the pathogenesis of NIID is still unknown, it shares many common features with another neurodegenerative disease‐Fragile X‐associated tremor ataxia syndrome (FXTAS). Both diseases involved the CNS and caused late‐onset pyramidal and extrapyramidal symptoms including cognitive impairments, tremor, ataxia, and autonomic dysfunction. NIID and FXTAS both have similar MRI abnormalities at the corticomedullary junction of the brain. FXTAS is caused by a high number of a CGG triplet repeat within the *FMR1* gene. Similarly, recent studies showed that familial and sporadic NIID were associated with GGC repeat expansion in the *NOTCH2NLC* gene.^5^, ^6^, ^7^ These common features between NIID and FXTAS suggest that NIID may share a similar pathological mechanism to FXTAS. Different from FXTAS, NIID is not only involved in CNS, but also in the peripheral nervous system and other systems. Recently, the rapidly increasing case reports revealed the involvement of other systems besides CNS and patients with NIID demonstrated different systemic symptoms.^3^, ^9^, ^10^, ^11^ Since the disease has been named NIID, many clinicians pay more attention to the symptoms of CNS, for example, according to the symptoms in the CNS, the patients with familial NIID were divided into three subgroups: muscle weakness‐dominant type, Parkinsonism‐dominant type, and dementia‐dominant type^6^; however, other system symptoms may be ignored. It is still unknown whether eosinophilic intranuclear inclusions, the hallmark of NIID, are distributed in different systems and cause multiple systemic symptoms and signs.

In this study, we systematically analyzed 51 patients diagnosed with NIID and demonstrated that NIID is a multiple‐system disease by its clinical symptoms, signs, auxiliary examination, and the staining of ubiquitin/p62 (markers of eosinophilic intranuclear inclusions) in different tissues.

## Materials and Methods

### Ethical statement

A clinical study titled as the pathogenesis, clinical features, and treatment of NIID (No. XYFY2019‐KL059‐01) was approved by the Ethics Review Committee of the Affiliated Hospital of Xuzhou Medical University. All the tissue samples and sections were obtained from the Department of Pathology, the Affiliated Hospitals of Xuzhou Medical University (Xuzhou City, China). Written informed consent was obtained from all patients.

### Patient inclusion and exclusion

For the suspected NIID patients who had abnormal intensity signal at corticomedullary junction in DWI sequences of MRI with or without neurological symptoms, the peripheral blood was collected for genetic tests. As described previously,^3^ GGC repeats in the *NOTCH2NLC* gene were examined. Once abnormal GGC repeats in the *NOTCH2NLC* gene in the probands were confirmed, clinical data and peripheral blood of the family members of those probands were further collected. All the patients and their family members who had abnormal GGC repeats in the *NOTCH2NLC* gene were included. A total of 99 individuals were recruited in this study. Individuals who could not provide or refuse to provide detailed clinical data and physical examination were excluded in this study. Among these patients, 48 patients with NIID were excluded for the lack of clinical data. Therefore, a total of 51 patients were included in this study for further analysis.

### Genetic test for GGC repeats in the* NOTCH2NLC* gene

Peripheral blood was collected and DNA was extracted. Fluorescent PCR‐capillary gel electrophoresis method was used to determine the GGC repeats in the* NOTCH2NLC* gene as described previously.^3^ We defined expanded GGC repeats as pathogenic when it is more than 60 according to a previous study.^3^

### Clinical information collection

All the patients with abnormal expanded GGC repeats in the *NOTCH2NLC* gene were revisited and clinical data were collected from inpatient information or the visitation. A systematic physical examination was performed to assess mental status, neurological symptoms, and different systemic symptoms. All patients were recommended to complete brain MRI, abdominal ultrasound, pulmonary CT, biochemical tests, urine routine and thyroid functions, cerebrospinal fluid (CSF) examination, and electromyography.

### Immunohistochemistry staining

For immunohistochemical staining, an immunohistochemistry kit (C506333‐0050, Sangon Biotech, Shanghai, China) was used. Tissues embedded in paraffin were cut for sections at 5‐μm thickness. After the sections were de‐waxed and washed, antigen‐retrieval was performed by using citrate buffer (10 mmol/L, pH 6.0). The sections were then incubated with primary antibodies overnight. The primary antibodies used were monoclonal anti‐ubiquitin (ab7780; 1:100, Abcam Biosciences, Cambridge, MA) and anti‐p62 antibodies (ab91526; 1:150, Abcam Biosciences). On the next day, sections were incubated with horseradish peroxidase‐labeled secondary antibody and stained with 3,3'‐diaminobenzidine. Images were acquired by a digital microscope (DM1000; Leica, Wetzlar, Germany).

### Electron microscopy

Samples for electron microscopy were sliced into 1 × 1 × 1 mm^3^ size and fixed in 2.5% glutaraldehyde solution. Further, the samples were incubated in 1% osmium tetroxide, dehydrated with graded acetone, and embedded with resin. Ultrathin sections (50 nm) were cut with an ultramicrotome (Leica Micro‐Systems, UC‐7, Wetzlar, Germany). After 3% uranyl acetate and lead nitrate double staining, sections were examined and photographed under a transmission electron microscope (JEM‐1400 Plus).

## Results

### Genetic analysis confirmed NIID diagnosis

**Figure 1:**
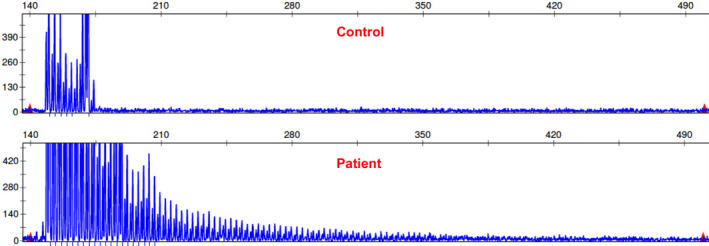
GGC repeats in the NOTCH2NLC gene from patients with NIID and control subjects. The peripheral blood samples were collected and DNA was extracted. Repeat‐primed PCR was performed to examine the GGC repeats in the NOTCH2NLC gene.

In this study, the total 51 patients from 12 families and five sporadic individuals were diagnosed with NIID based on abnormal GGC repeats in the *NOTCH2NLC* gene (Fig. 1). Among these patients, there were 27 male patients and 24 female patients aging from 13 to 79 years old (median: 63 years old).

### Clinical symptoms, signs, and auxiliary examinations showed that multiple systems were involved in NIID

Surprisingly, except neurological symptoms, clinical manifestations from other systems were very notable and diverse in 51 NIID patients. The proportions of different system symptoms were listed as followings: nervous system (88.2%) > respiratory system (78.4%) > circulatory system (72.5%) = locomotor system (72.5%) > urinary system (66.7%) > digestive system (64.7%) > reproductive system (61.5%) > endocrine system (50.0%). In addition, other common symptoms also included sexual dysfunction (43.1%), pupil constriction (56.9%), blurred vision (51.0%), and hearing loss (23.5%). Therefore, we summarized clinical findings from different systems based on symptoms, signs, laboratory, and pathological findings to further demonstrate that NIID is a multiple‐system disease (Table 1 and Table S1).

**Table 1:** The clinical evidence and initial symptoms characteristics of SIID.

Systems	Clinical evidence		Auxiliary examination evidence		Pathological tissues				Initial symptoms			
	No.	Core symptoms	No.	Core positive result	Cell type	UB	p62	Tissue source (case No.)	No.	Case No.	Median (year)	
Nervous system	45	Cognitive dysfunction, tremor, paroxysmal encephalopathy, neurogenic bladder	37	Zigzag edging sign, brain atrophy, white matter lesions and peripheral neuropathy	Brain	＋＋	＋＋＋	Brain (26), PNS (2, 6, 8, 23, 43, 46)	11	7, 9, 10, 20, 26, 28, 36, 38, 43, 44, 48	50
					Ganglia	＋＋＋	＋＋＋				
					Ganglia	＋＋	＋＋				
					Ganglia	＋＋	＋＋				
					Ganglia	＋＋	＋＋				
					Ganglia	＋＋	＋＋＋				
					Ganglia	＋	＋				
Respiratory system	32	Intractable dry cough	34	Chronic inflammation, nodules, interstitial changes, and emphysema	Lung	＋＋	＋＋	Lung (2)	19	3, 4, 5, 8, 11, 12, 13, 14, 15, 21, 22, 24, 32, 33, 34, 41, 42, 45, 49	42
Circulatory system	24	Valvular regurgitation, postural hypotension, paroxysmal chest distress	37	Valve regurgitation, cardiac insufficiency, T wave or ST‐T changes	Lymph gland	＋＋	＋＋	Lymph glands (2 and 43), blood vessel (1, 2, 6, 8, 23, 25, 43, 31, 46, 50）	4	1, 18, 37, 39	38.5
					Lymph gland	＋	＋＋				
					Blood vessel	＋	＋				
					Blood vessel	＋＋	＋＋				
					Blood vessel	＋＋	＋＋				
					Blood vessel	＋＋	＋＋				
					Blood vessel	＋＋	＋＋				
					Blood vessel	＋	＋＋				
					Blood vessel	＋＋	＋＋				
					Blood vessel	＋＋	＋＋＋				
					Blood vessel	＋＋	＋＋＋				
					Blood vessel	＋＋	＋＋				
Locomotor system	36	Joint pain, weakness	18	Joint degeneration and ligament injury	–	1	27	3			
Urinary system	27	Increased urinary frequency, urgency, incontinence, neurogenic bladder, urinary infection	30	Thickening bladder wall, kidney cyst, urinary infection, and renal insufficiency	Kidney	＋	＋＋	Kidney (1), urinary bladder (31, 46, 50)	6	6, 23, 30, 35, 46, 51	55
					Bladder	＋	＋＋				
					Bladder	＋＋	＋＋				
					Bladder	＋	＋＋＋				
Digestive system	27	Nausea, vomiting, constipation, gastrointestinal polyps	26	Hypohepatia, intrahepatic nodules, and gastrointestinal polyps	Esophagus	＋	＋＋	Esophagus (6), stomach (2, 27, 31, 44, 47), colon (1, 2, 25, 44), rectum (2, 40, 43), appendix (40), gallbladder (6)	7	2, 19, 25, 31, 40, 47, 50	30
					Stomach	＋＋＋	＋＋＋				
					Rectum	＋＋＋	＋＋＋				
					Sigmoid colon	＋＋	＋＋				
					Stomach	＋	＋				
					Sigmoid colon	＋	＋＋				
					Transverse colon	＋＋	＋＋				
					Sigmoid colon	＋＋	＋＋				
					Stomach	＋	＋				
					Descending colon	＋＋＋	＋＋＋				
					Rectum	＋	＋＋＋				
					Stomach	–	–				
					Stomach	–	–				
					Rectum	＋＋	＋				
					Appendix	＋＋	＋				
					Gallbladder	＋＋	＋＋＋				
Reproductive system	15	Male: Benign Prostatic Hyperplasia. Female: irregular vaginal bleeding, and infertility	16	prostatic hyperplasia and uterine leiomyomas	Prostate	＋＋＋	＋＋＋	Prostate (30, 31), fallopian tube (17), uterus (13), breast (39)	2	16, 17	28.5
					Prostate	＋	＋＋				
					Fallopian tube	＋	＋				
					Uterus	＋＋＋	＋				
					Breast	–	–				
Endocrine system	12	Hyperglycemia, abnormal thyroid function	21	Abnormal blood glucose and thyroid function	–	–	–	–			
Unclassified	–	Behavior disorder, personality changes, Sexual dysfunction, and miosis		Skin	＋＋＋	＋＋＋	Skin (6, 12, 23, 46, 50, 51)				
				Skin	＋＋＋	＋＋					
				Skin	＋＋	＋＋					
				Skin	＋＋	＋＋					
				Skin	–	–					
				Skin	＋	＋					

### Nervous system symptoms and evidences

The evidence of symptoms, signs, and auxiliary examinations supported the involvement of the nervous system in about 88.2% patients. Core symptoms included abnormal behaviors (60.8%, 31 cases), cognitive dysfunction (58.8%, 30 cases), Parkinsonism (51.0%, 26 cases), peripheral nervous symptoms (43.1%, 22 cases), and encephalopathy (21.6%, 11 cases). Some symptoms and signs such as pupil constriction (56.9%, 29 cases) and symptoms of neurogenic bladder (23.5%, 12 cases) were considered the involvement of autonomic nervous system.

**Figure 2:**
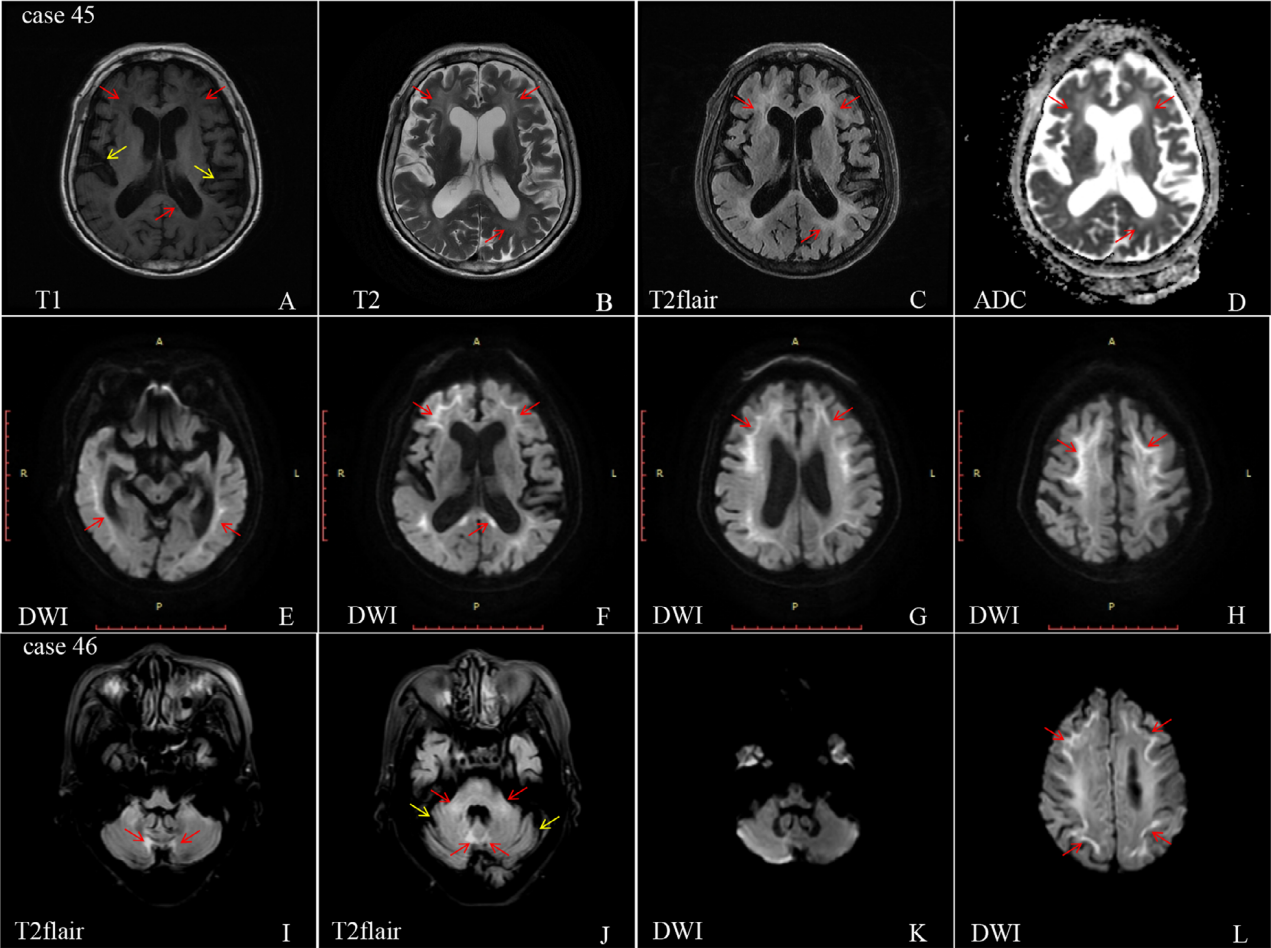
Typical features of MRI in patients with NIID. A representative case (case 45, A–H) shows the typical imaging manifestations showed T1 low signals (red arrows) and high signals in T2, FLAIR, and ADC axial images involving the corticomedullary junction of frontal lobe, parietal lobe, occipital lobe, temporal lobe, and corpus callosum (A–D). DWI shows ribbon signs imaging manifestations involving the corticomedullary junction, accompanied by cerebral atrophy, mainly in the temporal lobe and hippocampus (E–H). Another representative case (case 46, I–L) showed bilateral high signal intensity in the medial part of the cerebellar hemisphere immediately beside the vermis (the paravermal area, I) and in the middle cerebellar peduncle (red arrows, J) as well as the atrophy of the cerebellum (yellow arrows, J). DWI axial images showed no obvious abnormality in the cerebellum (K); however, obvious high signal intensity was found along the corticomedullary junction (L).

**Figure 3:**
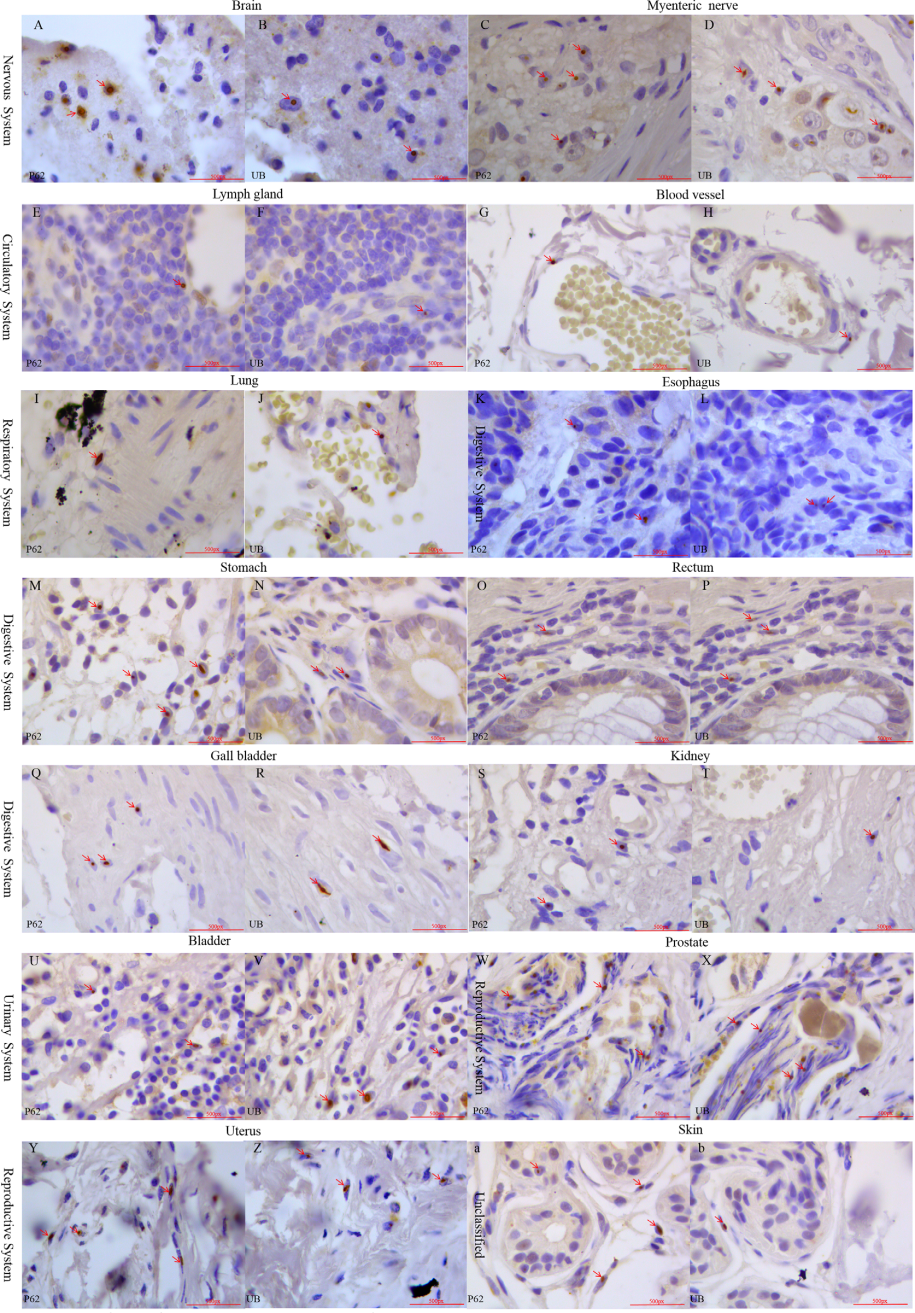
Positive immunostaining of anti‐p62 and anti‐ubiquitin in different tissues. The tissue samples obtained from the previous surgery in patients with NIID were stained with anti‐p62 and anti‐Ubiquitin antibodies. Immunohistochemical staining in nervous (A–D), circulatory (E–H), digestive (I–P), urinary (Q–T), reproductive (U–X), respiratory (Y and Z) systems, and skin tissues (a and b).

A total of 40 patients examined head CT and/or MRI. Most patients had different extents of brain atrophy (Fig. 2A and J, 37 out of 40 patients). Thirty‐six patients performed MRI examination. Except for three normal head MRIs, others had abnormal MRI findings (91.7%). Most patients (75.7%) revealed abnormal intensity signals in corticomedullary junction and periventricular regions in different sequences of MRI including (T1WI, T2WI, T2flair, and ADC, Fig. 2A–D, red arrow). The typical sign was a zigzag edging sign in corticomedullary junction in the DWI sequence of MRI (Fig. 2E–H and L, red arrow). In addition, abnormal high‐intensity signals in T2flair sequence were also observed in the callosum (Fig. 2F, 30 out of 36 patients) and the cerebellum or middle peduncle (Fig. 2I and J, 20 out of 36 patients). CSF examination was performed in four patients. CSF protein level was significantly increased, while cell counts, glucose and chloride levels of CSF were normal; CSF IgG and IgM were significantly increased (two to 10‐fold and more). Brain biopsy in one case showed focal tissue liquefactive necrosis surrounding with foam cell aggregation and glial hyperplasia in the right frontal lobe, which was similar to the pathology of post‐infarction. However, anti‐p62 and anti‐ubiquitin positive staining was observed in the nuclei of neurons around the lesion in brain cortex (Fig. 3A and B), myenteric nerve (Fig. 3C and D), and peripheral ganglion (six cases, Table 1).

### Respiratory system symptoms and evidences

About 78.4% of patients had respiratory system symptoms. Common symptoms were intractable irritant dry cough (51.0%, 26 cases). Chest CT was performed in 34 cases with a positive rate of 89.5% including chronic inflammation (45.5%), nodules (33.3%), interstitial changes (24.2%), or emphysema (15.2%) in the lung. Lung biopsy showed normal alveolus structure, but telangiectasia and infiltration of neutrophils monocytes in pulmonary interstitial. Ubiquitin and p62‐positive staining were found in lung tissues (Table 1, Fig. 3Y and Z).

### Circulatory system symptoms and evidences

Evidences supported the involvement of circulatory system in about 72.5% patients. Common symptoms were paroxysmal chest pain (35.3%, 18 cases) and postural hypotension (29.4%, 15 cases). Electrocardiogram showed T wave or ST‐T changes in 11 cases, atrioventricular block in four cases, and atrial or ventricular premature beats in four cases in the total 30 patients. Heart ultrasonic Doppler indicated valve regurgitation (21 cases, 84.0%), cardiac insufficiency (20 cases, 80.0%) in 25 patients. Ubiquitin and p62‐positive staining were found in blood vessels (Fig. 3E and F) from 10 cases including mesocolic lymph nodes (Fig. 3G and H), skin, gallbladder, colon, rectum, bladder, and prostate (Table 1 and Table S1).

### Locomotor system symptoms and evidences

About 72.5% patients had symptoms of locomotor system. Core clinical manifestations were muscle weakness (62.7%, 32 cases) and joint pain (39.2%, 20 cases). Joint and spine MRI/CT showed joint degeneration and joint and/or ligament injury in 18 patients (100%, 18 cases).

### Urinary system symptoms and evidences

Evidences supported the involvement of urinary system in 66.7% patients. Core clinical manifestations were frequent and urgent urination (49.0%, 25 out of 51 cases). Renal function insufficiency was found in five (11.1%) out of 45 cases. Routine urine test indicated 15 patients (36.6%) had urinary tract infection in 41 cases. Ultrasonic Doppler, CT, or MRI findings indicated thickening bladder wall (55.0%), kidney cyst (45.0%), hydronephrosis (20.0%), ureteral dilation (20.0%), and excess residual urine (30.0%) in 12 patients. Bladder biopsy in three cases showed diffused inflammatory cell infiltration and edema (Table 1). Anti‐p62 and anti‐ubiquitin‐positive cells were observed in kidney and bladder tissues (Fig. 3Q–T).

### Digestive system symptoms and evidences

**Figure 4:**
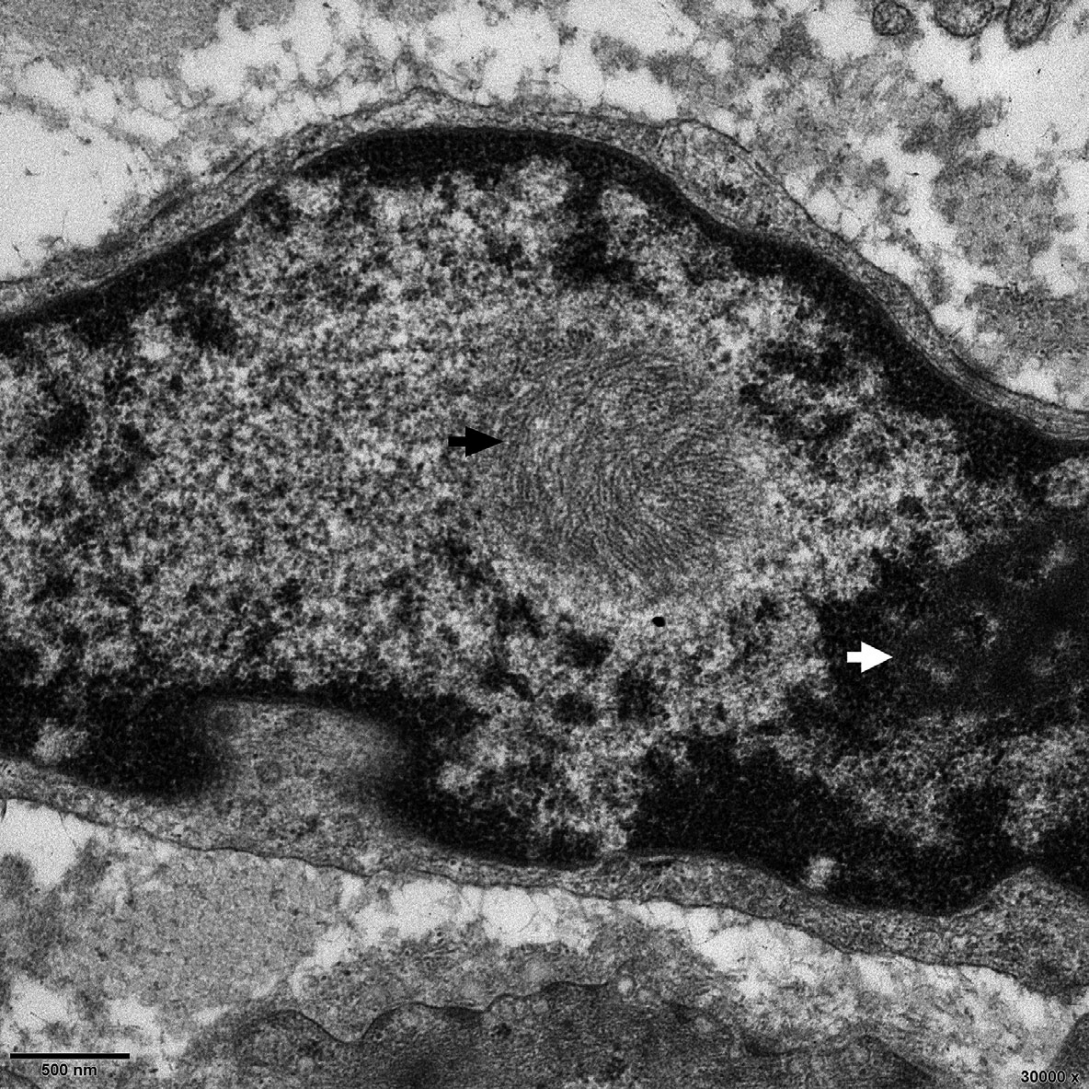
A representative case (patient 6) showed an intranuclear inclusion (black arrow) near a nucleolus (white arrow) in rectum tissues under an electron microscope.

About 64.7% patients had digestive system symptoms. Core clinical symptoms were constipation (47.1%, 24 cases) and nausea/vomiting (43.1%, 22 cases). Seven patients (15.6%) had abnormal liver functions in 45 patients. Liver ultrasonic Doppler or CT showed positive findings in all 24 cases including intrahepatic nodules (10 cases, 37.0%) and gallbladder wall roughness or stones (nine cases, 33.3%). Gastrointestinal polyps were found in 10 cases (83.3%) by gastroenteroscopy in 12 patients. Esophagus, stomach, gallbladder, colon, and rectum biopsy showed inflammatory cell infiltrate, tissue necrosis, and focal edema (Table 1). Ubiquitin and p62‐positive cells in different tissues were shown in Fig. 3I–P as examples. Electron microscopy showed the aggregates in the nucleus in rectum tissues (Fig. 4).

### Reproductive system symptoms and evidences

About 61.5% patients had reproductive system symptoms. Common symptoms in males were the symptoms of benign prostatic hyperplasia such as dysuria and dribbling urination; the common symptoms in females were irregular vaginal bleeding and infertility. Ultrasonic Doppler showed prostatic hyperplasia in male patients (six cases out of 12 patients) and myoma of uterus in female patients (seven cases out of eight patients). Prostate biopsy in two cases showed diffused inflammatory cell infiltration and gland hyperplasia (Table 1). Ubiquitin and p62‐positive cells were shown in prostate and uterus tissues (Fig. 3U–X).

### Endocrine system symptoms and evidences

Evidences supported the low involvement of endocrine system in about 50.0% patients for insidious symptoms. Core manifestations were hyperglycemia‐ and hypothyroidism‐related symptoms including polydipsia, polyuria, and emaciation, lower‐extremity edema (15.7%, eight cases). Ten cases had high glycosylated hemoglobin (HbA1c ≥6.2%) in 33 patients. Also, the thyroid function test confirmed 10 cases with hypothyroidism in 33 patients.

### Other system symptoms and evidences

Other symptoms such as blurred vision (51.0%), sexual dysfunction (43.1%), hearing loss (23.5%), and skin ulcers (13.7%) were very common in patients. Skin biopsy was performed in six cases (Table 1). Ubiquitin and p62 staining in skin were positive in five cases as shown in Figure 3A and B.

### Initial symptoms and median onset age in different systems indicated system heterogeneity of NIID

**Figure 5:**
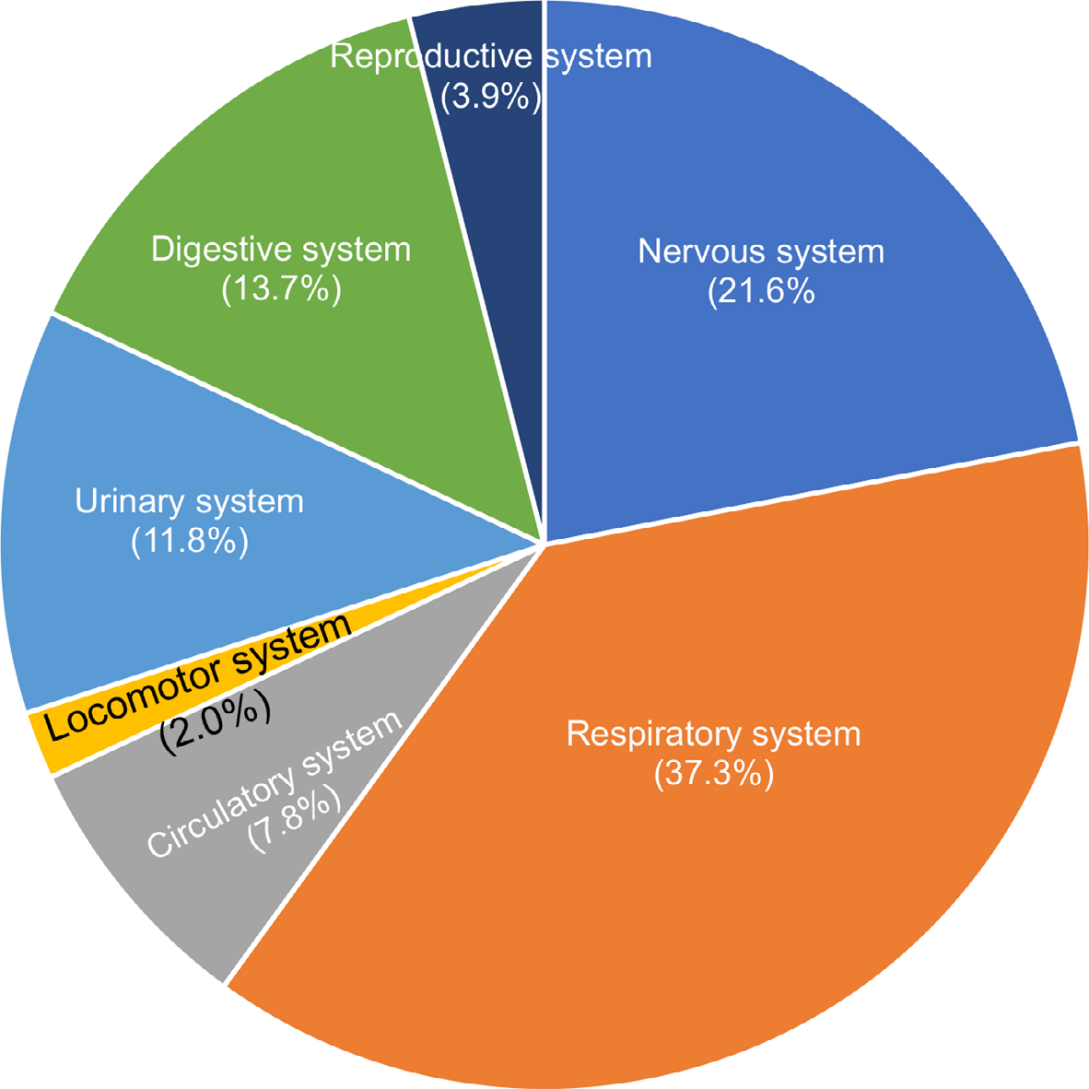
The diagram showed the percentages of initial symptoms from different systems. Initial symptoms in the total 51 patients with NIID were summarized.

The percentage of initial symptoms of NIID in different systems was listed as followings: respiratory system (37.3%) > nervous system (21.6%) > digestive system (13.7%) > urinary system (11.8%) > circulatory system (7.8%) > reproductive system (3.9%) > locomotor system (2.0%). Median onset age in different systems was 3 years in locomotor system, 28.5 years in reproductive system, 30 years in digestive system, 38.5 years in circulatory system, 42 years in respiratory system, 50 years in nervous system, and 55 years in urinary system (Table 1, Table S1 and Fig. 5).

## Discussion

### NIID is a systemic intranuclear inclusion disease (SIID)

NIID was described as a neurodegenerative disease that mainly involved the brain^3^; however, increasing lines of evidences have established that NIID was often accompanied with symptoms from different systems.^3^, ^9^, ^10^, ^11^ In this study, we are the first time to systemically summarize the clinical symptoms in NIID patients that were confirmed by genetic tests. Nervous system symptoms are main manifestations for NIID, but in our patients, there were still 11.8% patients with no nervous system symptom and signs; therefore, we could not view a symptom spectrum of other system involvement as concomitant symptoms of NIID. Actually, in our patients, we observed symptoms and signs from almost all the systems (Table 1), especially, the popular symptoms from respiratory system, digestive system, circulatory system, and urinary system. Except for symptoms, signs, or auxiliary examination, we also provided pathological evidences in 24 patients to show that intranuclear inclusion existed in all the systems by using surgical pathological tissues to examine the staining of ubiquitin and p62. To our knowledge, it is the first time to show the multiple system involvements of intranuclear inclusion in NIID by the large sample size of pathological tissues. Therefore, our evidence indicates that SIID is more appropriate to describe the nature of the disease than the term NIID.

### Time sequence of the affected system symptoms in NIID

In most previous reported cases, only the diagnosis was suspected for the patients with neurological symptoms and neuroimaging, skin biopsy was considered to perform for the pathological support.^2^, ^3^, ^12^ Skin biopsy was regarded a useful diagnostic tool for NIID and the deposits of intranuclear inclusion in skin was a diagnostic hallmark for NIID.^2^ However, our cases and other reports also showed that the presence of intranuclear inclusion in skin was later than that in other systems such as kidney and bladder or the occurrence of neurological symptoms^10^, ^13^, ^14^; indicating that positive skin involvement is not necessary for the early diagnosis of NIID. In our study, some symptoms of other systems preceded the symptoms of nervous system. For example, only 11 cases (21.6%) initial symptoms were the symptoms of nervous system (Table 1); initial symptoms in respiratory system (19 cases, 37.3%) had a higher proportion than that in the nervous system. In addition, the onset age of initial symptoms in different systems was much younger than that in the nervous system. Symptoms in locomotor system such as joint pain, limb weakness, or fatigue were the earliest initial symptoms in patients with NIID. These findings demonstrate that CNS involvement was in the late stage of NIID. Therefore, the physicians should pay more attention to early symptoms in other systems and perform genetic tests for the diagnosis of NIID.

### SIID is a symptom‐heterogeneous disease

Because of symptom heterogeneity of nervous system, NIID were clinically classified into different subgroups in different studies such as dementia, Parkinsonism, limb weakness, or dyskinesia.^3^, ^6^ In this study, we found that many NIID patients had different system symptoms at same time such as limb weakness, dry cough, persistent nausea, and late stage dementia. Different NIID families had different phenotypes from multiple systems; even in same family, different familiar members showed different symptoms with other members. Therefore, highly variable and no‐specific clinical symptoms make SIID be a high heterogeneous disease and hard to diagnosis. Recent advances on genetic studies facilitated the early diagnosis for SIID by detecting the repeat number of the *NOTCH2NLC* gene.^5^, ^6^, ^7^ Unlike Huntington disease or spinocerebellar ataxia, the affected patients with expanded CAG trinucleotide repeats have anticipation in the next generations^15^, ^16^; NIID patients with expanded GGC repeats in *NOTCH2NLC* did not show anticipation in the younger generation. This may be related with the fact that expanded GGC repeats in the* NOTCH2NLC* gene locate in the noncoding region in the NOTCH2NLC protein.^7^ However, anticipation was observed in familiar essential tremor patients with expanded GGC repeats in the *NOTCH2NLC* gene.^17^ Therefore, this needs more investigations in larger sample populations.

In this study, some symptoms reported are frequent in the general population in particular age groups such as frequent urination and urgent urination in elderly males as a result of benign prostatic hyperplasia. Therefore, as one of limitation of this study, we have no evidence to demonstrate that these symptoms are indeed directly related to the intranuclear inclusions of NIID. However, these symptoms were found to happen in the younger age and to be more severe in NIID patients; for example, frequent urination and urgent urination were commonly found in young NIID patients and prolonged use of indwelling catheters was common in elderly NIID patients; 23.5% NIID patients were diagnosed to have neurogenic bladder, which is much higher than the incidence in normal population. Therefore, our observations indicate that these common symptoms described here may be related to the pathology of NIID. This needs to be verified in a larger sample size of NIID patients.

In summary, for the first time, we systematically demonstrated that NIID is a heterogeneous and systemic neurodegenerative disease by providing clinical and pathological evidence in a large sample of patients. The symptoms from locomotor system, reproductive system or digestive system are much earlier than the symptoms from nervous system. Therefore, due to the disease heterogeneity, genetic tests should be provided for the suspected patients for the early diagnosis of NIID.

## Author Contributions

CH, CG, LK, RHK, SS, BL, and ZR collected and analyzed clinical data; CH, WX, WY, and MY performed immunohistochemistry staining. LL, WB, WB, and SM performed genetic tests; XX, CH, and SM designed the study and wrote the manuscript.

## Conflict of Interest

All authors report no disclosures.

## Data Availability Statement

Data are available upon reasonable request.

## Supporting information


**Table S1.** The summary of clinical symptoms in 51 patients with NIID.
