# Supplementary material for: Re‐defining the clinicopathological spectrum of neuronal intranuclear inclusion disease
Source: Ann Clin Transl Neurol. 2020 Sep 15;7(10):1930–41. doi: 10.1002/acn3.51189 (PMC7545592; doi:10.1002/acn3.51189)
Supplement: Supplementary file 1 — Table S1. The summary of clinical symptoms in 51 patients with NIID. [file ACN3-7-1930-s001.docx]

**Supplemental Table 1.** The summary of clinical symptoms in 51 patients with NIID

| Families | No. | Gender | Age(Y) | Clinical features | | | | | | | | | | | | | | | | Unclassified | | | | | Initial system | Age of onset (Y) | Course of disease (Y) | | Follow-up | |  |  |
| --- | --- | --- | --- | --- | --- | --- | --- | --- | --- | --- | --- | --- | --- | --- | --- | --- | --- | --- | --- | --- | --- | --- | --- | --- | --- | --- | --- | --- | --- | --- | --- | --- |
|  |  |  |  | NS | | RS | CS | | LS | | US | | DS | | RS | RPS | | ES | | SD | BV | Miosis | | HL |  |  |  |  |  |  |  |  |
|  |  |  |  | SS | AE | SS | AE | SS | AE | SS | AE | SS | AE | SS | AE | SS | AE | SS | AE | SS | AE | |  |  |  |  |  |  | |  | |  |
| Family 1 | case1 | F | 70 | Y | Y | N | Y | Y | Y | Y | Y | Y | Y | Y | Y | — | — | Y | N | Y | Y | | Y | Y | Y | Y | CS | 20 | | 50 | | yes |
|  | case2 | M | 68 | Y | Y | Y | Y | Y | Y | Y | Y | Y | N | Y | Y | — | — | N | Y | Y | Y | | Y | Y | Y | Y | DS | 15 | | 53 | | yes |
|  | case3 | M | 61 | Y | Y | Y | Y | Y | Y | Y | — | Y | Y | N | Y | Y | Y | Y | Y | Y | Y | | Y | Y | Y | Y | RS | 20 | | 41 | | yes |
| Family 2 | case4 | F | 78 | Y | Y | Y | Y | N | — | Y | — | N | N | N | N | — | — | N | — | — | — | | — | — | Y | Y | RS | 48 | | 30 | | dead |
|  | case5 | F | 70 | Y | Y | Y | Y | N | Y | Y | — | Y | Y | Y | N | — | — | Y | Y | — | N | | Y | Y | Y | Y | RS | 35 | | 35 | | yes |
|  | case6 | M | 67 | Y | Y | Y | Y | Y | Y | Y | — | Y | Y | Y | Y | Y | Y | N | N | Y | Y | | Y | Y | Y | Y | US | 35 | | 32 | | yes |
|  | case7 | F | 63 | Y | Y | Y | Y | Y | Y | Y | — | Y | Y | Y | N | — | — | Y | Y | Y | Y | | Y | Y | Y | Y | NS | 55 | | 8 | | yes |
|  | case8 | F | 56 | Y | N | Y | — | N | — | N | — | Y | — | Y | Y | — | — | N | — | Y | Y | | Y | N | Y | N | RS | 26 | | 30 | | yes |
|  | case9 | M | 50 | Y | N | N | — | N | — | N | — | N | — | N | — | — | — | — | — | — | — | | — | — | Y | N | NS | 4 | | 46 | | no |
|  | case10 | F | 64 | Y | Y | Y | Y | Y | Y | N | — | Y | N | — | Y | — | — | Y | N | — | Y | | Y | Y | Y | Y | NS | 60 | | 4 | | Dead |
|  | case11 | M | 63 | Y | Y | Y | Y | Y | Y | N | — | N | — | Y | N | Y | Y | N | N | — | N | | Y | N | Y | Y | RS | 23 | | 40 | | yes |
|  | case12 | M | 62 | Y | Y | Y | Y | Y | Y | Y | — | Y | Y | N | N | Y | Y | N | Y | Y | Y | | Y | N | Y | Y | RS | 45 | | 17 | | yes |
|  | case13 | F | 56 | Y | Y | Y | — | Y | Y | N | — | N | Y | N | N | Y | Y | N | Y | Y | Y | | Y | N | Y | Y | RS | 30 | | 26 | | yes |
|  | case14 | M | 52 | Y | Y | Y | — | N | — | Y | — | N | — | N | — | — | — | N | — | — | — | | — | — | Y | Y | RS | 42 | | 10 | | yes |
|  | case15 | F | 49 | Y | — | Y | — | N | N | Y | Y | N | — | N | — | N | — | Y | Y | — | N | | N | N | Y | — | RS | 43 | | 6 | | yes |
|  | case16 | M | 36 | Y | — | N | — | N | — | Y | — | N | — | N | — | Y | Y | N | — | N | N | | N | N | Y | — | RPS | 31 | | 5 | | yes |
|  | case17 | F | 33 | N | N | N | — | N | — | N | — | N | — | N | — | Y | Y | N | — | N | N | | N | N | N | N | RPS | 26 | | 7 | | yes |
|  | case18 | M | 29 | N | N | N | — | Y | Y | N | — | N | — | N | — | N | — | N | — | N | N | | N | N | N | N | CS | 24 | | 5 | | yes |
|  | case19 | M | 35 | N | N | N | N | N | N | N | — | N | — | N | Y | N | — | N | — | N | N | | N | N | N | N | DS | 34 | | 1 | | yes |
| Family 3 | case20 | F | 66 | Y | Y | N | Y | Y | Y | Y | — | Y | — | Y | N | — | — | N | Y | — | — | | — | — | Y | Y | NS | 50 | | 16 | | no |
|  | case21 | M | 62 | Y | Y | Y | Y | N | Y | Y | — | N | Y | N | Y | N | — | N | Y | N | Y | | Y | N | Y | Y | RS | 50 | | 12 | | yes |
| Family 4 | case22 | M | 67 | Y | Y | Y | Y | Y | Y | Y | Y | Y | Y | Y | Y | — | — | N | N | — | — | | Y | N | Y | Y | RS | 10 | | 57 | | Dead |
|  | case23 | M | 61 | Y | Y | N | Y | N | Y | Y | Y | Y | Y | Y | Y | — | — | N | Y | — | Y | | N | N | Y | Y | US | 41 | | 20 | | yes |
|  | case24 | M | 63 | Y | N | Y | Y | N | Y | N | — | N | — | N | — | N | — | N | — | N | Y | | N | — | Y | N | RS | 13 | | 50 | | yes |
|  | case25 | M | 46 | Y | N | N | Y | N | Y | Y | — | N | Y | Y | Y | N | — | N | Y | Y | Y | | Y | N | Y | N | US | 48 | | 13 | | yes |
| Family 5 | case26 | F | 66 | Y | Y | Y | Y | N | Y | Y | Y | Y | Y | Y | N | — | — | N | Y | Y | N | | Y | N | Y | Y | NS | 55 | | 11 | | Dead |
|  | case27 | M | 43 | N | — | Y | N | N | N | Y | Y | N | N | Y | Y | — | — | N | N | Y | N | | N | N | N | — | LS | 3 | | 40 | | yes |
|  | case28 | F | 44 | Y | N | Y | Y | Y | Y | N | — | Y | Y | N | N | N | Y | Y | Y | — | N | | N | Y | Y | N | NS | 1 | | 43 | | yes |
|  | case29 | F | 13 | N | N | N | — | N | — | N | — | N | — | N | — | N | — | N | — | — | N | | N | N | N | N | no | — | | — | | yes |
| Family 6 | case30 | M | 72 | Y | Y | N | N | — | Y | Y | — | — | Y | — | Y | Y | Y | — | Y | — | Y | | — | — | Y | Y | US | 62 | | 10 | | Dead |
|  | case31 | M | 65 | Y | Y | Y | Y | Y | Y | Y | — | Y | Y | Y | Y | Y | Y | N | N | Y | Y | | Y | Y | Y | Y | DS | 30 | | 35 | | yes |
| Family 7 | case32 | M | 64 | Y | Y | Y | Y | Y | Y | Y | Y | N | Y | N | N | — | — | Y | Y | Y | Y | | Y | N | Y | Y | RS | 45 | | 19 | | yes |
|  | case33 | F | 60 | Y | Y | Y | Y | Y | Y | Y | Y | Y | Y | N | N | N | — | Y | Y | N | N | | Y | N | Y | Y | RS | 45 | | 15 | | yes |
|  | case34 | M | 54 | Y | Y | Y | Y | Y | Y | Y | Y | Y | Y | Y | N | — | — | N | N | Y | Y | | Y | N | Y | Y | RS | 42 | | 10 | | yes |
| Family 8 | case35 | F | 59 | Y | Y | N | — | Y | Y | Y | — | Y | Y | Y | N | Y | Y | N | N | Y | Y | | Y | Y | Y | Y | US | 50 | | 9 | | no |
|  | case36 | F | 37 | Y | Y | N | — | N | — | N | — | N | — | N | — | — | — | N | — | — | N | | — | N | Y | Y | NS | 30 | | 7 | | no |
| Family 9 | case37 | M | 84 | Y | — | Y | Y | Y | Y | Y | — | Y | Y | N | — | Y | Y | N | N | — | N | | N | — | Y | — | CS | 76 | | 5 | | no |
|  | case38 | F | 67 | Y | Y | Y | — | N | N | Y | Y | N | N | Y | Y | — | — | N | Y | — | Y | | Y | N | Y | Y | NS | 35 | | 32 | | no |
|  | case39 | F | 63 | Y | Y | N | Y | N | Y | Y | Y | — | Y | Y | Y | Y | Y | N | N | — | — | | — | — | Y | Y | CS | 53 | | 10 | | no |
|  | case40 | M | 42 | N | — | N | — | N | — | N | — | N | — | Y | Y | N | — | N | — | — | — | | — | — | N | — | DS | 39 | | 3 | | no |
|  | case41 | M | 68 | Y | Y | Y | Y | Y | Y | Y | — | N | — | Y | Y | — | — | N | Y | — | N | | — | N | Y | Y | RS | 48 | | 20 | | yes |
|  | case42 | M | 67 | Y | Y | Y | Y | N | N | Y | Y | Y | Y | N | N | — | — | N | N | — | — | | — | — | Y | Y | RS | 47 | | 20 | | no |
| Family10 | case43 | M | 62 | Y | Y | Y | Y | Y | Y | N | Y | Y | Y | Y | Y | Y | Y | N | — | Y | N | | Y | N | Y | Y | NS | 57 | | 5 | | yes |
|  | case44 | M | 62 | Y | Y | — | Y | Y | Y | N | — | — | Y | Y | Y | Y | Y | N | N | — | — | | — | — | Y | Y | NS | 60 | | 2 | | no |
| Family11 | case45 | F | 74 | Y | Y | Y | Y | N | Y | Y | — | Y | Y | Y | Y | — | N | N | Y | Y | Y | | Y | Y | Y | Y | RS | 35 | | 39 | | yes |
| Family12 | case46 | F | 72 | Y | Y | N | Y | N | Y | Y | Y | Y | Y | Y | Y | — | — | N | N | Y | Y | | Y | N | Y | Y | US | 60 | | 12 | | Dead |
| Possible sporadic cases | case47 | F | 73 | Y | Y | N | N | Y | Y | Y | Y | N | N | Y | Y | N | N | N | Y | — | Y | | Y | Y | Y | Y | DS | 55 | | 18 | | yes |
|  | case48 | F | 79 | Y | Y | Y | Y | N | Y | Y | — | Y | Y | Y | Y | — | — | Y | N | Y | Y | | Y | N | Y | Y | NS | 15 | | 64 | | yes |
|  | case49 | F | 67 | Y | Y | Y | Y | N | Y | Y | Y | Y | Y | N | Y | Y | Y | Y | Y | Y | Y | | Y | N | Y | Y | RS | 35 | | 32 | | yes |
|  | case50 | F | 63 | Y | Y | N | Y | N | Y | Y | Y | Y | Y | Y | Y | — | — | Y | N | Y | Y | | Y | N | Y | Y | DS | 13 | | 50 | | yes |
|  | case51 | M | 70 | Y | Y | Y | Y | Y | Y | Y | — | Y | Y | N | N | — | — | N | N | Y | Y | | Y | N | Y | Y | US | 60 | | 10 | | yes |
| Positive rate(%) | | M/F= 9:8 | 63 | 88.2 | 72.5 | 62.7 | 66.7 | 47.1 | 72.5 | 70.6 | 35.3 | 52.9 | 58.8 | 52.9 | 51.0 | 62.7 | 66.7 | 29.4 | 31.4 | 23.5 | 41.1 | | 43.1 | 51 | 56.9 | 23.5 | RS(19) | 37.5 | | 22.7 | | 80.4 |
| Total positive rate(%) | | |  | 88.2 | | 78.4 | | 72.5 | | 72.5 | | 66.7 | | 64.7 | | 78.4 | | 61.5 | | 50 | | | 43.1 |  |  |  |  |  |  |  |  |  |

Abbreviations: NS, nervous system; RS, respiratory system; CS, circulatory system; LS, locomotor system; US, urinary system; DS, digestive system; RPS, reproductive system; ES, endocrine system; SS, the existence of symptoms or signs; AE, positive auxiliary examination; No, number; SD, sexual dysfunction; BV, blurred vision; HL, hearing loss; Y, yes; N, no; -, not available.
